# Seroprevalence Study of Anti-HEV IgG among Different Adult Populations in Corsica, France, 2019

**DOI:** 10.3390/microorganisms7100460

**Published:** 2019-10-16

**Authors:** Lisandru Capai, Shirley Masse, Pierre Gallian, Cécile Souty, Christine Isnard, Thierry Blanchon, Brigitte Peres, Xavier de Lamballerie, Rémi Charrel, Alessandra Falchi

**Affiliations:** 1 EA 7310, Laboratoire de Virologie, Université de Corse, 20250 Corte, France; masse_s@univ-corse.fr; 2 Etablissement Français du Sang Provence alpes Côte d’Azur et Corse, 13005 Marseille, France; pierre.gallian@efs.sante.fr (P.G.); Christine.ISNARD@efs.sante.fr (C.I.); Brigitte.PERES@efs.sante.fr (B.P.); 3 Unité des Virus Émergents (UVE), Aix Marseille Univ, IRD 190, INSERM 1207, IHU Méditerranée Infection, 13005 Marseille, France; xavier.de-lamballerie@univ-amu.fr (X.d.L.); remi.charrel@univ-amu.fr (R.C.); 4 Sorbonne Université, INSERM, Institut Pierre Louis d’épidémiologie et de Santé Publique (IPLESP UMRS 1136), F-75012 Paris, France; cecile.souty@iplesp.upmc.fr (C.S.); thierry.blanchon@iplesp.upmc.fr (T.B.)

**Keywords:** seroprevalence, hepatitis E, IgG, risk factor, corsica, hyperendemic, hepatitis, France

## Abstract

Hepatitis E virus (HEV) is a major cause of acute hepatitis worldwide. In France, hyperendemic areas including Corsica have an anti-HEV Immunoglobulin G (IgG) prevalence higher than 50%. The aim of this study was to determine the seroprevalence of anti-HEV IgG in three adult populations in Corsica and the risk factors associated with antibody detection. Between 2017 and 2019, a total of 930 individuals, including 467 blood donors, 393 students or university staff members and 70 patients from general practice, were tested for the presence of anti-HEV IgG using the Wantai HEV IgG enzyme immunoassay kit and filled a questionnaire. The association between seropositivity and potential risk factors was tested with univariate and multivariate analyses. Out of the 930 samples, 52.3% (486/930) were seropositive—54.4% (254/467) among blood donors, 47.6% (187/393) among university students and 64.3% (45/70) among patients of general practice. Three main risk factors were identified: (i) skinning and butchering (Adjusted Odds Ratio aOR = 2.76, 95% confidence interval [95% CI] [1.51–5.37]; *p*-value < 10^−3^), (ii) consumption of a local pork live raw sausage (fittonu) (aOR = 1.95 95% CI [1.45–2.64]; *p*-value = 10^−5^), and (iii) increasing age (*p*-value = 0.003). Seropositivity rates between the different populations were homogeneous after age stratification. This cross-sectional study indicates a high anti-HEV IgG seroprevalence in the Corsican adult population, not significantly different between women and men and increasing with age. This serosurvey also showed homogeneity regarding the exposure to HEV among three different types of populations. Finally, we confirmed the endemicity of Corsica with respect to HEV and identified a strong association between consumption of figatellu/fittonu and the practice of skinning and butchering with the detection of anti-HEV IgG.

## 1. Introduction

Hepatitis E virus (HEV) possesses a single-stranded positive-sense RNA genome of approximately 7.5 kb which contains three separate open reading frames (ORFs) [1,2]. This virus belongs to family *Hepeviridae* (genus *Orthohepevirus*) [3]. Viral strains infecting humans are classified into five genotypes (HEV-1 to HEV-4 and HEV-7) [4], but belong to a single serotype.

Genotypes HEV-3 and 4 are zoonotic pathogens infecting domestic (e.g., pigs) and wild animal species (e.g., boars, deers) which constitute the animal reservoir [5]. They are mainly detected in industrialized countries and are responsible for sporadic and autochthonous cases [1,6]. In Europe, the majority of cases is due to HEV-3 genotype and HEV is the most common cause of acute viral hepatitis [7].

A recent meta-analysis describing the HEV seroprevalence in industrialized countries, based on studies using the Wantai HEV Immunoglobulin G (IgG) enzyme immunoassay, reported an overall seroprevalence of 19% [14–25%], with great heterogeneity between countries and regions (<5% in New Zealand to >50% among French regions) [8].

The largest seroprevalence study conducted in France on 10,569 blood donors reported a 22.4% (95% [CI] 21.6–23.2%) rate with significant geographical differences and the identification of hyperendemic areas (prevalence >50%) in southern and northeastern regions [9]. The presence of anti-HEV IgG was associated with increasing age and the consumption of pork/pork liver sausages/wild game meat/offal/oysters. Conversely, drinking bottled water was associated with a lower rate of anti-HEV IgG [9]. A national report on the surveillance of HEV in France has highlighted a sharp increase (9 to 2292) in the number of autochthonous cases reported to the public health authorities between 2002 and 2016 [10]. This apparent increase is likely due (i) to improved diagnostic tests and (ii) to better awareness among physicians and in the general population, resulting in increased testing rather than a true epidemic situation. However, seroprevalence data (rates >50%), the hospitalization rate per 100,000 inhabitants, and the total number of prescribed serological tests underline the hyperendemicity of HEV in southern France.

In Corsica, a French Mediterranean island, the seroprevalence was estimated at 62% [9]. The main risk factor observed in Corsica seems to be the consumption of raw pig liver sausage (figatellu [plural: figatelli]; small liver in the Corsican language) which is traditionally eaten grilled. Indeed, grouped cases of HEV have been described and related to its consumption [11,12]. Pavio et al. (2014) described the presence of HEV RNA in 30% of tested figatelli. In Corsica, other behaviors may correlate with higher exposure to HEV such as the frequent practice of hunting, the consumption of food products derived from the porcine reservoir (figatellu and fittonu) and the existence of important rural areas. 

Except for specific groups of patients (immunodepressed patients, transplanted patients, etc.), the population of blood donors is the largest group for which HEV seroprevalence studies have been performed [13,14]. Here, we recruited two additional populations to evaluate whether exposure to HEV was similar or different in other adult populations. 

There is a lack of in-depth information about the actual impact of transmission route links with the porcine reservoir and other alternative sources of contamination (including environmental sources) on the epidemiology of HEV in Corsica. The main objective of this study was to improve the knowledge about the epidemiology of HEV in Corsica island, using a seroprevalence study including a large cohort of adults consisting of blood donors, general practitioner patients and staff and students of the University of Corsica. The present study gives new insights into the epidemiology of HEV in an endemic area of metropolitan France.

## 2. Materials and Methods

### 2.1. Ethics

The study received approval from the medical and scientific direction of the French Public Transfusion service (Établissement Français du Sang: EFS) and from the ad hoc ethics committee (Comité de Protection des Personnes #2016-A01000-51, 11 January 2017). The questionnaire and all data collected were validated by the data protection officer of the University of Corsica (UCPP). All participants were included on a voluntary non-remunerated basis. They were informed that samples will be used for seroprevalence studies by a letter of information and they signed a consent form. 

### 2.2. Study Populations

Participants were included in the study if they declared living in Corsica for at least six months, at enrolment. 

Population A Blood donors (BD): Voluntary blood donors accepted for classical donation according to the national requirements and agreeing to complete the questionnaire were included from 11 March 2019 to 15 April 2019.

Population B University of Corsica Pascal Paoli (UCPP): Students and personnel of the UCPP were included from January 2017 to January 2019 on the different campuses of the UCPP. 

Population C Patient from General Practice (PGP): Patients from general practice >18 years old were recruited by General Practitioners (GPs) from June 2017 to September 2017. All participants were informed about the study by letter or during a face-to-face discussion with a member of the health staff.

### 2.3. Questionnaire

The questionnaire contained information about socio-demographical variables (age, gender, educational level, professional activities, type of dwelling, and sewage disposal), clinical factors (presence of chronic diseases, transplantation, blood transfusion, immunosuppression, and a past HEV infection during the life of the individual), contact with animals (pets and/or domestic farm animals), the consumption of meat (big game, little game, pork, beef, poultry, giblets, and pork liver), derived meat products (figatellu, fittonu, pâté/terrine, and sausages), fish and shellfish (seafood), organic fruits and vegetables or personal vegetable garden, wild berries and the source of drinking water (bottled, tap, mountain water sources, and fountains). We also recorded the type of cooking levels (raw, rare, medium, and well cooked) (items listed in Appendix A). Only the UCPP and PGP populations were asked as to clinical factors (presence of chronic diseases, transplantation, blood transfusion, immunosuppression, and a past HEV infection during the life of the individual).

The survey was conducted in the presence of knowledgeable medical personnel to ensure the accuracy of data collection. 

### 2.4. Blood Samples and Laboratory Methods

#### 2.4.1. Blood Samples

The blood samples obtained from blood donors corresponded to EDTA (Ethylenediamine tetraacetic acid) tubes collected systematically during the standard protocol. Samples from the UCPP and PGP groups were from capillary blood and were obtained using a safety lancet on a cleansed puncture finger that was collected into 0.8 mL tubes containing a coagulation activator and serum separator; these tubes were centrifuged at 6000 rpm for 15 min and the resulting serum was stored at −20 °C until processed for serology.

#### 2.4.2. Anti-HEV IgG Detection

Serum samples were analyzed for the presence of anti-HEV IgG (EFS Provence-Alpes-Côtes-d’Azur & Corse, Marseille, and Laboratoire de Virologie Université de Corse) using the Wantai HEV IgG enzyme immunoassay kit (Wantai Biologic Pharmacy Enterprise, Beijing, PRC). The assay is based on a recombinant antigen corresponding to open reading frame 2 [15], the analytical and clinical performances of which were evaluated recently with a specificity and sensitivity of 97.96% and 99.60%, respectively [16]. Analyses were performed according to the manufacturer’s instructions. For each sample, the ratio (sample OD/cutoff OD) was calculated and values ≥1 were positive. This assay was chosen in order to be in line with our previous work and to compare our results with the main French serosurvey [9] and the majority of European seroprevalence studies. In addition, this test is used by the National Reference Center for Hepatitis E in France.

### 2.5. Statistical Analysis 

#### 2.5.1. Sample Size

The sample size was calculated according to previously described methods [17]. A sample size of 384 was calculated assuming an a priori 50% anti-HEV seroprevalence, a confidence in the estimate of 95%, a maximum allowable error in the prevalence of 5%, and a Corsican population size of 330,455 habitants (based on the latest French census data).

#### 2.5.2. Seroprevalence and Epidemiological Factors Analysis

Descriptive statistics were performed for all variables. Continuous data were reported as medians with interquartile ranges (IQRs). All categorical data were reported as percentages. 

HEV seroprevalence (IgG) and its 95% exact binomial confidence intervals (CIs) were estimated for each population and overall. Frequencies were compared using the χ^2^ test or Fisher’s exact test (*p*-value < 0.05).

Associations between explanatory variables (socio-demographic, lifestyle factors and eating habits) and having anti-HEV IgG were tested in univariate analyses for each population and overall. All variables with a *p*-value below 0.2 were included in the multivariate analyses using an unconditional logistic regression model. Statistical significance was set at a *p*-value <0.05. We also performed a logistic regression model with a random effect at the population level, taking into account that the people included came from different subpopulations. We used R packages (questionr, stats and lme4-package) and function glmer and glm. All statistical analyses were performed using the R program [18].

## 3. Results

**Figure 1:**
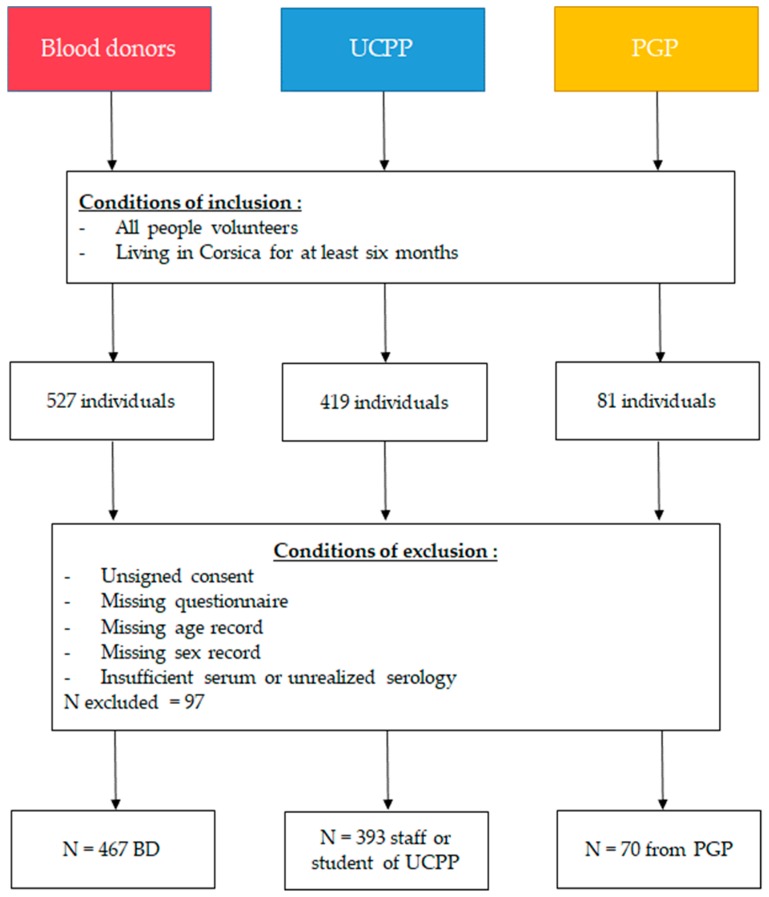
Flowchart for the inclusion and exclusion of studied populations.

A total of 930 individuals were included in the study: 467 BD, 393 UCPP, and 70 PGP (Figure 1). The characteristics of the three groups are presented in Table A1.

The overall median age was 32 years (IQR: 22–49). The median age of BD was 38 years (IQR: 28–52), 24 years (IQR: 20–35) for UCPP and 55 years (IQR: 44–67) for GP. Age and gender distributions differed significantly when we compared the three groups.

Among the PGP and UCPP groups, no participant was declared to have knowledge of previous infection due to HEV (0/463).

### 3.1. Anti-HEV IgG Seroprevalence

Anti-HEV IgG were detected in 54.4% (*n* = 254) (IC 95% [49.8–58.9]) of the BD group, in 47.6% (*n* = 187) (IC 95% [42.6–52.5]) of the UCPP group and in 64.3% (*n* = 45) (IC 95% [53.1–75.5]) of the PGP group (Table 1). Prevalence differed significantly among the three populations (*p*-value = 0.015).

Males had higher rates compared with females in each of the three groups, but significantly higher only in BD and globally (*p*-value = 0.00169 and 0.00009, respectively) (Table 1).

**Figure 2:**
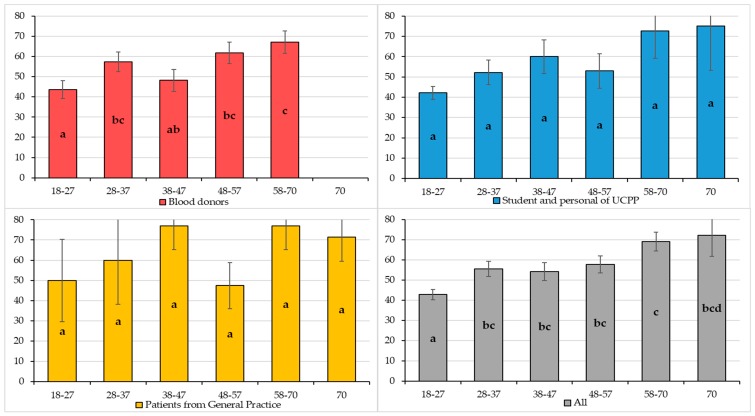
Graphs of the different seroprevalences of anti-HEV IgG (%) by age group and populations. The different lowercase letter (a, b, c, and d) indicates a significant difference (*p* < 0.05) between age groups of a given population. For example: among blood donors, the group 18–27 is significantly different from that of 58–70 (“a” vs. “c”), but not from that of 38–47 (presence of the letter a). The black bars correspond to the standard deviation of each proportion.

Seroprevalence according to age groups and to population groups is presented in Figure 2. Among the UCPP and PGP populations, there was no significant difference between the different age groups (same letter). In BD, seroprevalences of the youngest age group (18–27) and the oldest (58–70) were significantly different (*p*-value < 0.05 illustrated by letters a and c). Globally, the seroprevalence of the youngest age group (18–27) was significantly different compared with all other age groups. Between the 28 to 57 years age groups, there was a plateau with observed rates that were very close to each other (54.2% to 57.7%; letter b in common), followed by two higher seroprevalences of 69.1% and 72.2% for the oldest age groups (58–70 and > 70 years) (Figure 2). For a given age group, the seroprevalence rates are not significantly different between the three groups (all *p*-values > 0.05).

### 3.2. Risk Factors Associated with Anti-HEV IgG Seroprevalence

#### 3.2.1. Univariate Analysis

Results of univariate analyses and seroprevalences by variable are presented in Table 1.

In the BD group, fourteen variables were significantly associated with higher seropositivity rates (*p*-value < 0.05): age groups, male (OR = 1.81 [1.25–2.64]), breeding (OR = 2.79 [1.15–7.78]), skinning and butchering (OR = 3.05 [1.07–10.88]), fountain water (OR = 1.58 [1.08–2.33]), mountain spring waters (OR = 1.76 [1.2–2.6]), big wild game (OR = 1.8 [1.23–2.65]), pork (OR = 2.95 [1.36–6.92]), sausages and pâtés (OR = 2.47 [1.37–4.62]), liver (OR = 1.82 [1.2–2.77]), figatellu (OR = 4.14 [2.37–7.53]), fittonu (OR = 2.6 [1.76–3.86]), offal (OR = 1.86 [1.25–2.78]) and wild berries (OR = 1.57 [1.07–2.31]).

In the UCPP group, the exposure for ten variables presented significantly higher seroprevalences: hunting (OR = 2.24 [1.17–4.45]), skinning and butchering (OR = 2.73 [1.37–5.75]), big wild game (OR = 1.84 [1.19–2.88]), pork (OR = 3.26 [1.35–9.09]), sausages and pâtés (OR = 2.6 [1.11–6.78]), liver (OR = 1.56 [1.01–2.42]), figatellu (OR = 2.71 [1.53–4.98]), fittonu (OR = 2.24 [1.47–3.42]), offal (OR = 1.78 [1.15–2.76]) and shellfish (OR = 1.98 [1.17–3.45]).

In the PGP group, eight variables were significantly associated with a higher anti-HEV IgG detection rate: hunting (OR = INF), skinning and butchering (OR = INF), tap water (OR =3.66 [1.14–12.58]), mountain spring waters (OR = 2.89 [1.01–9.21]), big wild game (OR = 3.14 [1.1–9.25]), fittonu (OR = 4.08 [1.46–12.47]), beef (OR = 8.38 [1.15–169.46]) and wild berries (OR = 3.38 [1.21–10.26]).

Only three variables were significantly associated with anti-HEV IgG in the three populations: skinning/butchering, big wild game, and consumption of fittonu.

The mean increase in seroprevalence for the practice of skinning and butchering is +25% (the increases ranged between +16% and +40% according to population) and +23% for consumption of fittonu (the increases ranged between +20% and +31% according to population). People who reported eating big game had a higher seroprevalence of 20% compared to those who did not eat it (45.1% vs. 59.7% among BD; 36.6% vs. 51.5% among UCPP and 45.5% vs. 72.3% among PGP).

Cooking types (overall population values in the Table A2) and clinical factors (data not shown) were not associated with anti-HEV IgG-seropositivity (*p*-value > 0.05) overall and among each population.

#### 3.2.2. Multivariate Analysis

In multivariate analysis (Table 2), “skinning and butchering” and “consumption of fittonu” remained independent predictors for anti-HEV IgG seropositivity in each population. Male and consumption of figatellu were associated with anti-HEV IgG detection only in the BD population (*p*-value = 0.03 and < 10^−3^ respectively).

In overall multivariate analyses (glm and lmer) “skinning and butchering” (aOR = 2.76; 95% CI 1.51–5.37), “consumption of fittonu” (aOR = 1.95; 95% CI 1.45–2.64) and of “figatellu” (aOR = 2.22; 95% CI, 1.45–3.45) were associated with anti-HEV detection. Increasing age was also significantly associated with anti-HEV IgG detection in the overall multivariate analysis (*p*-value = 0.003) (Table 2).

#### 4. Discussion

In France, seroprevalence studies were conducted in blood donors and allowed the identification of different risk factors such as increasing age, hunting, consumption of pork liver sausages (figatellu), game meat offal and oysters [9,19,20]. Hyperendemic areas were identified in southwestern France (Occitanie), southeastern France (Provence-Alpes-Cotes-d’Azur), and Corsica. Our study is the first to address Corsican populations other than blood donors, using a specific questionnaire and a large regional sampling.

A total of 52.2% of 930 individuals were positive for the presence of anti-HEV IgG. Interestingly, results of a very similar order of magnitude were observed in each of the three groups. Although the three groups were different regarding age, sex, and socio-demographic data, they seem to be fairly homogeneous with regard to HEV. Indeed, for a given age group, no significant differences in seroprevalences were observed.

We observed an age-related increase in anti-HEV IgG in the 18–27 group (42.8%) and the older than 70 group (73.7%), reflecting a cumulative life-time exposure in agreement with previous studies performed in European populations [9,13,21,22,23]. This age-related increased rate could be associated with differences in dietary habits and other behaviors such as rare or absence of consumption of uncooked meat products, a shorter exposure time and lack of hunting and skinning/butchering in the younger group. Repeated exposure leading to infection/reinfection might also play a role, although it appears that a single infection leads to long-life immunity [24], and that there is no clear data about the possibility of immunity acquisition through repeated contacts with HEV without systemic infection. 

In the present study, although we recorded higher rates in men (59.8%) vs. women (46.9%), the difference was not statistically significant in the multivariate analysis. This is in line with previous studies performed in French blood donors living in the hyperendemic regions in southern France [25] and in other industrialized countries [22,26,27]. Overall, these results could suggest that exposure to HEV is not directly related to gender but rather to individual behavior (differences in dietary habits and other behaviors such as a different frequency of consumption of meat products, and lack of hunting or other practices in contact with the animal reservoir).

Interestingly, none of the 463 participants and, more specifically, none of the 232 participants with anti-HEV IgG (50.2%) reported a known previous infection with HEV. This suggests that asymptomatic cases or cases of infection for which the patients are not seeking medical check-up might be much larger than the 50–80% commonly reported for genotype 3 [28,29,30]. This is specifically important in hyperendemic regions where efforts should be exerted for better awareness of Hepatitis E and for a more systematic strategy of testing compared to what is currently done. The risk factors described in our study together with those reported in the scientific literature should be used to define “the at-risk population” which merit to be tested for the presence of viral RNA and for anti-HEV IgM whenever clinical manifestations are coherent with acute infection with HEV.

An independent factor associated with anti-HEV IgG seropositivity was to practice skinning or butchering. In Corsica, traditionally, hunter or breeder family members commonly engage in such activities, leading to HEV exposure in a distinct manner compared to food or water intake. This practice was strongly associated with HEV IgG positivity in our study. HEV infection can occur during the evisceration of an infected animal—through contact with its blood or feces. In a similar manner, higher rates in seroprevalence studies were identified in butchers and slaughterhouse workers compared with the general population [31]. The presence of HEV RNA in wild boar and swine bile, liver, sera, and faces [11,32,33] is in line with a higher risk of exposure and strengthens the need for protective gloves during the disemboweling of wild boars [34].

Here, we observed that participants reporting eating specific types of meat such as figatellu were significantly associated with higher HEV IgG seropositivity as previously described [35,36,37]. Our study was the first to examine the consumption of fittonu, a dried pork liver sausage that is not cooked before eating (in contrast with figatellu). In the Netherlands, traditional Dutch dry raw sausages called “cervelaat”, “fijnkost”, “salami” and “salametti” were also associated with higher seroprevalence [38]. Interestingly, anti-HEV IgG rates were in range from 27% to 31% according to three recent Dutch studies [8,22,38,39]. The same situation is observed in Poland with seroprevalence between 44% and 50% and where Polish dry sausage known as “Kabanos” are very popular [8].

A cooking temperature of 71 °C for twenty minutes is required to inactivate HEV [40,41]. The virus stays viable after heating at 56 °C for one hour and remains infectious up to 60 °C [41,42]. Although figatellu is usually roasted, it remains strongly associated with HEV infection; cooking does not appear to have a significant impact on seroprevalence in our study (*p*-value = 0.87). This could be explained by the fact that figatellu is also (i) either eaten without being roasted or (ii) eaten after the necessary cooking times and temperatures have not been respected; (iii) last, pre-roasting handling of the raw figatellu might be a risk [43]. In this regard, washing hands after product handling or wearing gloves during disemboweling must be recommended.

Meat products were not statistically associated with HEV seropositivity but showed higher rates (>55%) (game, offal, liver, pork). These meat products have been frequently associated with higher seroprevalence or HEV RNA detection [44,45,46,47]. 

HEV is increasingly found in the environment [48]. In our study, higher anti-HEV IgG rates were associated with consumption of fountain waters in villages and waters of natural springs (mountain hiking), and seafood. In Corsica, the Regional Health Agency (Agence Régionale de la Santé) has carried out a study on the quality of drinking water, and many counties reported unsatisfactory bacteriological results although HEV was not tested [49]. As a non-enveloped virus, HEV transmission through water consumption (such as hepatitis A virus and other picornaviruses) must be taken into consideration as an important route of infection. Many studies have identified the presence of HEV in running water. In Italy, HEV was detected in river water [50]. Irrigation water is also involved, and the virus was deteted in fruits and vegetables in several European countries [51,52,53,54] or associated with higher seroprevalence in Turkey [55]. 

HEV was repeatedly detected in seafood in the United Kingdom, Spain and Japan [56,57,58,59] or epidemiologically associated with higher anti-HEV IgG rates in population with frequent consumption of seafood and shellfish [9]. In these studies, strains belonging to genotype 3 (swine and human strains) were identified, suggesting the existence of an epidemiologic cycle between the different animal reservoir, environment, and human cases. 

This study has several limitations. First, we investigated anti-HEV IgG seroprevalence in the adult population only. Data for children are scarce, and the risk factors remain to be thoroughly evaluated [60]. Second, the sample issued from PGP was small in size with respect to the samples issued from blood donors and university. Third, we cannot exclude that other risk factors that seem to play a minor role in our study may be more prominent when increasing the sample size. Finally, the number of individuals included who were older than 70 years was small compared to other age groups, which may underestimate the overall seroprevalence calculated.

This cross-sectional study indicates an anti-HEV IgG seroprevalence > 50% in the Corsican adult population, similar between women and men and increasing with age. This serosurvey also showed homogeneity regarding the exposure to HEV among three different types of populations. Finally, we identified a strong association between consumption of figatellu/fittonu and the practice of skinning and butchering, with the detection of anti-HEV IgG among the three populations studied. These results provide relevant information for control and preventive strategies and concrete advice to risk groups. Surface, irrigation, or consumption water could be a potential source for exposure. A study on the presence of the virus in surface waters or bivalve molluscan shellfish as an indicator of water pollution (or food products) could be carried out to better understand the epidemiology of the virus in Corsica.

## Figures and Tables

**Table 1:** Seroprevalence and factors associated with anti-Hepatitis E virus (HEV) IgG detection (univariate analysis). Significant *p*-values < 0.05 are followed by an asterisk.

**INF: Infinite**		**BD**					**UCPP**				
**NA: Missing Values**		***n***	**Anti-HEV IgG Positive**		**OR [95% CI]**	***p*-Value**	***n***	**Anti-HEV IgG Positive**		**OR [95% CI]**	***p*-Value**
**Parameters**	**Variables**		***n***	**%**				***n***	**%**		
**Gender**	Female	266	128	48.1	Reference	0.00169 *	244	107	43.9	Reference	0.05807
	Male	201	126	62.7	1.81 [1.25–2.64]		149	80	53.7	1.48 [0.99–2.24]	
**Age groups**	18–27	126	55	43.7	Reference	0.00633 *	242	102	42.1	Reference	0.07139
	28–37	101	58	57.4	1.74 [1.03–2.97]		67	35	52.2	1.5 [0.87–2.59]	
	38–47	83	40	48.2	1.2 [0.69–2.1]		35	21	60.0	2.06 [1.01–4.32]	
	48–57	84	52	61.9	2.1 [1.2–3.71]		34	18	52.9	1.54 [0.75–3.2]	
	58–70	73	49	67.1	2.64 [1.46–4.87]		11	8	72.7	3.66 [1.03–17.02]	
	70	NA					4	3	75.0	4.12 [0.52–83.9]	
**Hunting**	No	447	240	53.7	2.01 [0.79–5.77]	0.14486	350	159	45.4	2.24 [1.17–4.45]	0.01428 *
	Yes	20	14	70.0			43	28	65.1		
**Breeding**	No	442	235	53.2	2.79 [1.15–7.78]	0.02165 *	366	173	47.3	1.2 [0.55–2.66]	0.64555
	Yes	25	19	76.0			27	14	51.9		
**Skinning and butchering**	No	449	240	53.5	3.05 [1.07–10.88]	0.03549 *	354	160	45.2	2.73 [1.37–5.75]	0.00403 *
	Yes	18	14	77.8			39	27	69.2		
**Contact with wastewater**	No	450	243	54.0	1.56 [0.58–4.6]	0.37978	383	181	47.3	1.67 [0.47–6.64]	0.42524
	Yes	17	11	64.7			10	6	60.0		
**Tap water**	No	41	23	56.1	0.92 [0.47–1.74]	0.78775	25	11	44.0	1.16 [0.52–2.69]	0.71415
	Yes	410	221	53.9			360	172	47.8		
**Bottled water**	No	15	10	66.7	0.57 [0.17–1.63]	0.29725	10	5	50.0	0.88 [0.24–3.19]	0.835
	Yes	440	234	53.2			375	175	46.7		
**Water fountains in the villages**	No	213	102	47.9	1.58 [1.08–2.33]	0.01895 *	168	69	41.1	1.51 [1–2.29]	0.05103
	Yes	211	125	59.2			199	102	51.3		
**Mountain spring waters**	No	207	96	46.4	1.76 [1.2–2.6]	0.00401 *	182	86	47.3	0.96 [0.64–1.45]	0.83939
	Yes	212	128	60.4			184	85	46.2		
**Little wild game**	No	295	150	50.8	1.45 [0.97–2.18]	0.06953	227	100	44.1	1.34 [0.89–2.02]	0.16436
	Yes	145	87	60.0			154	79	51.3		
**Big wild game**	No	175	79	45.1	1.8 [1.23–2.65]	0.00264 *	123	45	36.6	1.84 [1.19–2.88]	0.00591 *
	Yes	268	160	59.7			260	134	51.5		
**Pork**	No	30	9	30.0	2.95 [1.36–6.92]	0.00579 *	26	6	23.1	3.26 [1.35–9.09]	0.0075 *
	Yes	421	235	55.8			356	176	49.4		
**Sausages and pâtés**	No	52	18	34.6	2.47 [1.37–4.62]	0.00263 *	26	7	26.9	2.6 [1.11–6.78]	0.027 *
	Yes	388	220	56.7			358	175	48.9		
**Liver**	No	301	147	48.8	1.82 [1.2–2.77]	0.00463 *	255	110	43.1	1.56 [1.01–2.42]	0.04589 *
	Yes	134	85	63.4			120	65	54.2		
**Figatellu**	No	69	18	26.1	4.14 [2.37–7.53]	2.46 × 10^−8^ *	65	18	27.7	2.71 [1.53–4.98]	0.00049 *
	Yes	374	222	59.4			322	164	50.9		
**Fittonu**	No	218	94	43.1	2.6 [1.76–3.86]	1.82 × 10^−6^ *	175	64	36.6	2.24 [1.47–3.42]	0.00015 *
	Yes	211	140	66.4			190	107	56.3		
**Offal**	No	281	136	48.4	1.86 [1.25–2.78]	0.00213 *	247	103	41.7	1.78 [1.15–2.76]	0.009 *
	Yes	159	101	63.5			125	70	56.0		
**Beef**	No	16	8	50.0	1.18 [0.43–3.27]	0.74187	15	9	60.0	0.58 [0.19–1.64]	0.3031
	Yes	430	233	54.2			368	171	46.5		
**Poultry**	No	16	8	50.0	1.18 [0.43–3.28]	0.73897	19	9	47.4	0.99 [0.39–2.56]	0.99112
	Yes	437	237	54.2			362	171	47.2		
**Shellfish**	No	76	38	50.0	1.2 [0.73–1.97]	0.47138	71	24	33.8	1.98 [1.17–3.45]	0.01107 *
	Yes	376	205	54.5			314	158	50.3		
**Fish**	No	22	10	45.5	1 [0.41–2.36]	0.99845	31	10	32.3	1.98 [0.93–4.51]	0.07726
	Yes	431	235	54.5			352	171	48.6		
**Organic fruits and vegetables**	No	62	27	43.5	1.64 [0.96–2.84]	0.07122	30	12	40.0	1.4 [0.66–3.07]	0.38191
	Yes	383	214	55.9			348	168	48.3		
**Wild berries**	No	234	115	49.1	1.57 [1.07–2.31]	0.02001 *	194	84	43.3	1.32 [0.88–1.99]	0.17687
	Yes	199	120	60.3			179	90	50.3		
**Total**		467	254	54.4			393	187	47.6		
		**PGP**					**Overall**				
		***n***	**Anti-HEV IgG Positive**		**OR [95% CI]**	***p*-Value**	***n***	**Anti-HEV IgG Positive**		**OR [95% CI]**	***p*-Value**
**Parameters**	**Variables**		***n***	**%**				***n***	**%**		
**Gender**	Female	34	20	58.8	Reference	0.35362	544	255	46.9	Reference	0.00009 *
	Male	36	25	69.4	1.59 [0.6–4.33]		386	231	59.8	1.69 [1.3–2.2]	
**Age groups**	18-27	6	3	50.0	Reference	0.40753	374	160	42.8	Reference	0.00001 *
	28-37	5	3	60.0	1.5 [0.13–19.11]		173	96	55.5	1.67 [1.16–2.4]	
	38-47	13	10	76.9	3.33 [0.43–29.22]		131	71	54.2	1.58 [1.06–2.37]	
	48-57	19	9	47.4	0.9 [0.13–5.99]		137	79	57.7	1.82 [1.23–2.71]	
	58-70	13	10	76.9	3.33 [0.43–29.22]		97	67	69.1	2.99 [1.87–4.87]	
	70	14	10	71.4	2.5 [0.34–19.67]		18	13	72.2	3.48 [1.28–11.02]	
**Hunting**	No	64	39	60.9	INF	0.01785 *	861	438	50.9	2.21 [1.32–3.82]	0.0024 *
	Yes	6	6	100.0			69	48	69.6		
**Breeding**	No	65	41	63.1	2.34 [0.32–47.23]	0.42697	873	449	51.4	1.75 [1.01–3.11]	0.04641 *
	Yes	5	4	80.0			57	37	64.9		
**Skinning and butchering**	No	61	36	59.0	INF	0.00322 *	864	436	50.5	3.07 [1.76–5.64]	0.00005 *
	Yes	9	9	100.0			66	50	75.8		
**Contact with wastewater**	No	67	42	62.7	INF	0.09878	900	466	51.8	1.86 [0.88–4.19]	0.1043
	Yes	3	3	100.0			30	20	66.7		
**Tap water**	No	15	6	40.0	3.66 [1.14–12.58]	0.02965 *	81	40	49.4	1.13 [0.71–1.78]	0.60849
	Yes	55	39	70.9			825	432	52.4		
**Bottled water**	No	2	2	100.0	INF	0.17973	27	17	63.0	0.62 [0.27–1.34]	0.22479
	Yes	68	43	63.2			883	452	51.2		
**Water fountains in the villages**	No	35	21	60.0	1.39 [0.52–3.8]	0.50842	416	192	46.2	1.5 [1.15–1.97]	0.00289 *
	Yes	34	23	67.6			444	250	56.3		
**Mountain spring waters**	No	42	23	54.8	2.89 [1.01–9.21]	0.04808 *	431	205	47.6	1.36 [1.04–1.79]	0.02331 *
	Yes	27	21	77.8			423	234	55.3		
**Little wild game**	No	46	27	58.7	1.99 [0.69–6.38]	0.20866	568	277	48.8	1.38 [1.05–1.82]	0.02055 *
	Yes	23	17	73.9			322	183	56.8		
**Big wild game**	No	22	10	45.5	3.14 [1.1–9.25]	0.03188	320	134	41.9	1.84 [1.4–2.43]	0.00001 *
	Yes	47	34	72.3			575	328	57.0		
**Pork**	No	1	0	0.0	INF	0.14871	57	15	26.3	3.27 [1.83–6.18]	0.00004 *
	Yes	69	45	65.2			846	456	53.9		
**Sausages and pâtés**	No	5	2	40.0	2.93 [0.45–23.52]	0.25158	83	27	32.5	2.44 [1.52–3.99]	0.00017 *
	Yes	65	43	66.2			811	438	54.0		
**Liver**	No	48	28	58.3	2.29 [0.75–7.92]	0.1474	604	285	47.2	1.7 [1.28–2.28]	0.00028 *
	Yes	21	16	76.2			275	166	60.4		
**Figatellu**	No	6	2	33.3	4.1 [0.74–31.28]	0.1066	140	38	27.1	3.48 [2.35–5.24]	1.88 × 10^−10^
	Yes	64	43	67.2			760	429	56.4		
**Fittonu**	No	35	17	48.6	4.08 [1.46–12.47]	0.00694 *	428	175	40.9	2.46 [1.87–3.24]	8.41 × 10^−11^
	Yes	34	27	79.4			435	274	63.0		
**Offal**	No	39	26	66.7	0.75 [0.28–2.02]	0.56837	567	265	46.7	1.72 [1.3–2.28]	0.00012 *
	Yes	30	18	60.0			314	189	60.2		
**Beef**	No	5	1	20.0	8.38 [1.15–169.46]	0.0349 *	36	18	50.0	1.08 [0.55–2.11]	0.82208
	Yes	65	44	67.7			863	448	51.9		
**Poultry**	No	2	1	50.0	1.83 [0.07–47.76]	0.67522	37	18	48.6	1.15 [0.59–2.24]	0.67789
	Yes	68	44	64.7			867	452	52.1		
**Shellfish**	No	15	7	46.7	2.49 [0.77–8.21]	0.12501	162	69	42.6	1.57 [1.11–2.21]	0.00988 *
	Yes	54	37	68.5			744	400	53.8		
**Fish**	No	3	1	33.3	3.83 [0.35–84.93]	0.26558	56	21	37.5	1.61 [0.94–2.83]	0.08474
	Yes	67	44	65.7			850	450	52.9		
**Organic fruits and vegetables or personal vegetable garden**	No	11	8	72.7	0.61 [0.12–2.38]	0.49248	103	47	45.6	1.34 [0.89–2.03]	0.1605
	Yes	58	36	62.1			789	418	53.0		
**Wild berries**	No	37	19	51.4	3.38 [1.21–10.26]	0.01937 *	465	218	46.9	1.52 [1.17–1.99]	0.00203 *
	Yes	32	25	78.1			410	235	57.3		
**Total**		70	45	64.3			930	486	52.3		

**Table 2:** Multivariate analysis in the three populations and overall.

		BD		UCPP		PGP		Overall Glm		Overall Lmer Fixed Subpopulations	
Parameters	Variables	aOR [95% CI]	*p*-Value	aOR [95% CI]	*p*-Value	aOR [95% CI]	*p*-Value	aOR [95% CI]	*p*-Value	aOR [95% CI]	*p*-Value
**Gender**	**Male**	1.58 [1.05–2.39]	0.02973	NS		NS		NS		NS	
**Figatellu**		2.82 [1.54–5.34]	0.00065					2.22 [1.45–3.45]	0.00023	1.77 [1.04–3.01]	0.035
**Fittonu**		1.97 [1.29–3.03]	0.00183	2.14 [1.4–3.28]	0.00039	4.35 [1.48–13.92]	0.00696	1.95 [1.45–2.64]	0.00001	1.95 [1.38–2.74]	0.00013
**Skinning and butchering**		3.52 [1.09–15.83]	0.03451	2.43 [1.16–5.38]	0.01764	7.88 e6 [0–INF]	0.00294	2.76 [1.51–5.37]	0.00077	3.45 [1.37–8.71]	0.0087
**Age groups**	**18–27**	NS		NS		NS		Reference	0.00272	NS	
	**28–37**							1.61 [1.09–2.38]			
	**38–47**							1.52 [0.98–2.37]			
	**48–57**							1.54 [1–2.38]			
	**58–70**							2.44 [1.45–4.19]			
	**70**							3.46 [1.17–12.65]			

NS: Non-significant value; INF: infinite value.

## Appendix A

List of items of the questionnaire

- Age
- Gender
- Situation
- Professional activity
- Educational level
- ZIP code
- Type of residence
- Water distribution
- Sewage disposal
- Contact with wastewater
- Consumption water
- Hunting
- Breeding
- Home at one kilometer of a farm or a breeding
- Skinning or butchering activity
- Contact with different animals
- Eating habits and type of cooking
- Clinical factors (presence of chronic diseases, transplantation, blood transfusion, immunosuppression, and a past HEV infection during the life of the individual) except for blood donors

**Table A1:** Description of population and repartition of individuals included by variable.

		Blood Donors		Student and Personal of UCPP		Patient of General Practice		Overall	
		*n*	%	*n*	%	*n*	%	*n*	%
**Gender**	**Men**	201	43.0	149	37.9	36	51.4	386	41.5
	**Women**	266	57.0	244	62.1	34	48.6	544	58.5
**Age groups**	**18–27**	126	27.0	242	61.6	6	8.6	374	40.2
	**28–37**	101	21.6	67	17.0	5	7.1	173	18.6
	**38–47**	83	17.8	35	8.9	13	18.6	131	14.1
	**48–57**	84	18.0	34	8.7	19	27.1	137	14.7
	**58–70**	73	15.6	11	2.8	13	18.6	97	10.4
	**70**			4	1.0	14	20.0	18	1.9
**Graduate studies level**	**Pre bac**	87	18.6	30	7.6	42	60.0	159	17.1
	**Bac**	145	31.0	180	45.8	8	11.4	333	35.8
	**Post bac**	232	49.7	179	45.5	20	28.6	431	46.3
**Professional activity**	**No**	64	13.7	112	28.5	1	1.4	177	19.0
	**Yes**	403	86.3	281	71.5	69	98.6	753	81.0
**Kind of habitation**	**Apartment**	179	38.3	182	46.3	23	32.9	384	41.3
	**Individual House**	284	60.8	204	51.9	47	67.1	535	57.5
	**Farm**	4	0.9	6	1.5	0	0.0	4	0.4
**Hunters**	**No**	447	95.7	350	89.1	64	91.4	861	92.6
	**Yes**	20	4.3	43	10.9	6	8.6	69	7.4
**Breeders**	**No**	442	94.6	366	93.1	65	92.9	873	93.9
	**Yes**	25	5.4	27	6.9	5	7.1	57	6.1
**Skinning and butchering**	**No**	449	96.1	354	90.1	61	87.1	864	92.9
	**Yes**	18	3.9	39	9.9	9	12.9	66	7.1
**Contact with wastewater**	**No**	450	96.4	383	97.5	67	95.7	900	96.8
	**Yes**	17	3.6	10	2.5	3	4.3	30	3.2
**Housing water**	**Private**	22	4.7	19	4.1	2	0.4	43	9.2
	**Collective**	429	91.9	360	91.6	69	98.6	858	92.3
**Total**		467	50.2	393	42.3	70	7.5	930	100.0

Missing values (NA): Seven for the variable “Graduate studies level” and “kind of habitation”; 29 for housing water.

**Table A2:** Univariate analysis of the association between anti-HEV IgG detection and the type of cooking.

Food Products	Cooking	OR [95% CI]	*p*-Value
**Little wild game**	Well-cooked	Reference	0.45337
	Raw	INF	
	Rare/Medium	0.85 [0.51;1.4]	
**Big wild game**	Well-cooked	Reference	0.23411
	Raw	INF	
	Rare/Medium	0.75 [0.5;1.15]	
**Pork**	Well-cooked	Reference	0.2255
	Raw	0.99 [0.4;2.49]	
	Rare/Medium	0.69 [0.46;1.05]	
**Sausages and pâtés**	Well-cooked	Reference	0.81779
	Raw	0.87 [0.53;1.45]	
	Rare/Medium	0.9 [0.56;1.44]	
**Liver**	Well-cooked	Reference	0.18698
	Raw	INF	
	Rare/Medium	0.71 [0.38;1.34]	
**Figatellu**	Well-cooked	Reference	0.86939
	Raw	1.18 [0.62;2.32]	
	Rare/Medium	1.05 [0.67;1.64]	
**Offal**	Well-cooked	Reference	0.17264
	Rare/Medium	0.65 [0.35;1.21]	
**Beef**	Well-cooked	Reference	0.60331
	Raw	1.29 [0.77;2.2]	
	Rare/Medium	1.01 [0.75;1.36]	
**Poultry**	Well-cooked	Reference	0.51125
	Raw	INF	
	Rare/Medium	0.97 [0.66;1.41]	
**Shellfish**	Well-cooked	Reference	0.40387
	Raw	1.24 [0.86;1.8]	
	Rare/Medium	1.3 [0.79;2.14]	
**Fish**	Well-cooked	Reference	0.7241
	Raw	1.07 [0.73;1.58]	
	Rare/Medium	1.16 [0.8;1.69]	

## References

[1] Kamar N., Bendall R., Legrand-Abravanel F., Xia N.S., Ijaz S., Izopet J., Dalton H.R. (2012). Hepatitis E. Lancet.

[2] Tam A.W., Smith M.M., Guerra M.E., Huang C.C., Bradley D.W., Fry K.E., Reyes G.R. (1991). Hepatitis E virus (HEV): molecular cloning and sequencing of the full-length viral genome. Virology.

[3] International Committee on Taxonomy of viruses Hepeviridae. https://talk.ictvonline.org/ictv-reports/ictv_online_report/positive-sense-rna-viruses/w/hepeviridae.

[4] Doceul V., Bagdassarian E., Demange A., Pavio N. (2016). Zoonotic hepatitis E virus: Classi fi cation, animal reservoirs and transmission routes. Viruses.

[5] Pavio N., Meng X.J., Renou C. (2010). Zoonotic hepatitis E: animal reservoirs and emerging risks. Vet. Res..

[6] Geng Y., Wang Y. (2016). Epidemiology of hepatitis E. Adv. Exp. Med. Biol..

[7] Adlhoch C., Avellon A., Baylis S.A., Ciccaglione A.R., Couturier E., de Sousa R., Epstein J., Ethelberg S., Faber M., Feher A. (2016). Hepatitis E virus: Assessment of the epidemiological situation in humans in Europe, 2014/15. J. Clin. Virol..

[8] Capai L., Falchi A., Charrel R. (2019). Meta-analysis of human IgG anti-HEV seroprevalence in industrialized countries and a review of literature. Viruses.

[9] Mansuy J.M., Gallian P., Dimeglio C., Saune K., Arnaud C., Pelletier B., Morel P., Legrand D., Tiberghien P., Izopet J. (2016). A nationwide survey of hepatitis E viral infection in French blood donors. Hepatology.

[10] Couturier E., Abravanel F., Figoni J., Van Cauteren D., Septfons A., Lhomme S., Durand J., Izopet J., De Valk H. (2018). Hepatitis E surveillance in France 2002–2016. 2018, 28, 566–574. Bull. Epidémiol. Hebd..

[11] Pavio N., Laval M., Maestrini O., Casabianca F., Charrier F., Jori F. (2016). Possible foodborne transmission of hepatitis E virus from domestic pigs and wild boars from corsica. Emerg. Infect Dis..

[12] Renou C., Roque-Afonso A.M., Pavio N. (2014). Foodborne transmission of hepatitis E virus from raw pork liver sausage, France. Emerg. Infect Dis..

[13] Lapa D., Capobianchi M.R., Garbuglia A.R. (2015). Epidemiology of hepatitis E virus in european countries. Int. J Mol Sci.

[14] Wilhelm B., Waddell L., Greig J., Young I. (2019). Systematic review and meta-analysis of the seroprevalence of hepatitis E virus in the general population across non-endemic countries. PLoS ONE.

[15] Zhang J., Ge S.X., Huang G.Y., Li S.W., He Z.Q., Wang Y.B., Zheng Y.J., Gu Y., Ng M.H., Xia N.S. (2003). Evaluation of antibody-based and nucleic acid-based assays for diagnosis of hepatitis E virus infection in a rhesus monkey model. J. Med. Virol..

[16] Avellon A., Morago L., Garcia-Galera del Carmen M., Munoz M., Echevarria J.M. (2015). Comparative sensitivity of commercial tests for hepatitis E genotype 3 virus antibody detection. J. Med. Virol..

[17] Arya R., Antonisamy B., Kumar S. (2012). Sample size estimation in prevalence studies. Indian J. Pediatr..

[18] R Core Team R: A Language and Environment for Statistical Computing, 2015. https://www.r-project.org/.

[19] Izopet J., Labrique A.B., Basnyat B., Dalton H.R., Kmush B., Heaney C.D., Nelson K.E., Ahmed Z.B., Zaman K., Mansuy J.M. (2015). Hepatitis E virus seroprevalence in three hyperendemic areas: Nepal, Bangladesh and southwest France. J. Clin. Virol..

[20] Mansuy J.M., Saune K., Rech H., Abravanel F., Mengelle C., L Homme S., Destruel F., Kamar N., Izopet J. (2015). Seroprevalence in blood donors reveals widespread, multi-source exposure to hepatitis E virus, southern France, October 2011. Eurosurveillance.

[21] Christensen P.B., Engle R.E., Hjort C., Homburg K.M., Vach W., Georgsen J., Purcell R.H. (2008). Time trend of the prevalence of hepatitis E antibodies among farmers and blood donors: a potential zoonosis in Denmark. Clin. Infect Dis..

[22] Slot E., Hogema B.M., Riezebos-Brilman A., Kok T.M., Molier M., Zaaijer H.L. (2013). Silent hepatitis E virus infection in Dutch blood donors, 2011 to 2012. Eurosurveillance.

[23] Olsoy I.B., Henriksen S., Weissbach F.H., Larsen M., Borgen K., Abravanel F., Kamar N., Paulssen E.J., Hirsch H.H., Rinaldo C.H. (2019). Seroprevalence of hepatitis E virus (HEV) in a general adult population in Northern Norway: the Tromso study. Med. Microbiol. Immunol..

[24] Zhou Y. (2016). Immunobiology and host response to HEV. Adv. Exp. Med. Biol..

[25] Mansuy J.M., Bendall R., Legrand-Abravanel F., Saune K., Miedouge M., Ellis V., Rech H., Destruel F., Kamar N., Dalton H.R. (2011). Hepatitis E virus antibodies in blood donors, France. Emerging Infect. Dis..

[26] Petrovic T., Lupulovic D., Jimenez de Oya N., Vojvodic S., Blazquez A.B., Escribano-Romero E., Martin-Acebes M.A., Potkonjak A., Milosevic V., Lazic S. (2014). Prevalence of hepatitis E virus (HEV) antibodies in Serbian blood donors. J. Infect Dev. Ctries..

[27] Pittaras T., Valsami S., Mavrouli M., Kapsimali V., Tsakris A., Politou M. (2014). Seroprevalence of hepatitis E virus in blood donors in Greece. Vox sanguinis.

[28] European Association for the Study of the Liver (2018). EASL clinical practice guidelines on hepatitis E virus infection. J. Hepatol..

[29] De Keukeleire S., Reynders M. (2015). Hepatitis E: An underdiagnosed, emerging infection in nonendemic regions. J. Clin. Transl. Hepatol..

[30] Xin S., Xiao L. (2016). Clinical manifestations of hepatitis E. Adv. Exp. Med. Biol..

[31] Teixeira J., Mesquita J.R., Pereira S.S., Oliveira R.M., Abreu-Silva J., Rodrigues A., Myrmel M., Stene-Johansen K., Overbo J., Goncalves G. (2017). Prevalence of hepatitis E virus antibodies in workers occupationally exposed to swine in Portugal. Med. Microbiol. Immunol..

[32] Jori F., Laval M., Maestrini O., Casabianca F., Charrier F., Pavio N. (2016). Assessment of domestic pigs, wild boars and feral hybrid pigs as reservoirs of hepatitis E virus in Corsica, France. Viruses.

[33] Capai L.F.C., Maestrini O., Villechenaud N., Masse S., Bosseur F., De Lamballerie X., Charrel R., Falchi A. (2019). Drastic decline of hepatitis E virus detection in domestic pigs after the age of 6 months, Corsica, France. Transbound. Emerg. Dis..

[34] Schielke A., Ibrahim V., Czogiel I., Faber M., Schrader C., Dremsek P., Ulrich R.G., Johne R. (2015). Hepatitis E virus antibody prevalence in hunters from a district in Central Germany, 2013: a cross-sectional study providing evidence for the benefit of protective gloves during disembowelling of wild boars. BMC Infect. Dis..

[35] Berto A., Grierson S., Hakze-van der Honing R., Martelli F., Johne R., Reetz J., Ulrich R.G., Pavio N., Van der Poel W.H., Banks M. (2013). Hepatitis E virus in pork liver sausage, France. Emerg. Infect. Dis..

[36] Colson P., Borentain P., Queyriaux B., Kaba M., Moal V., Gallian P., Heyries L., Raoult D., Gerolami R. (2010). Pig liver sausage as a source of hepatitis E virus transmission to humans. J. Infect. Dis..

[37] Pavio N., Merbah T., Thebault A. (2014). Frequent hepatitis E virus contamination in food containing raw pork liver, France. Emerg. Infect. Dis..

[38] Mooij S.H., Hogema B.M., Tulen A.D., van Pelt W., Franz E., Zaaijer H.L., Molier M., Hofhuis A. (2018). Risk factors for hepatitis E virus seropositivity in Dutch blood donors. BMC Infect. Dis..

[39] Van Gageldonk-Lafeber A.B., van der Hoek W., Borlee F., Heederik D.J., Mooi S.H., Maassen C.B., Yzermans C.J., Rockx B., Smit L.A., Reimerink J.H. (2017). Hepatitis E virus seroprevalence among the general population in a livestock-dense area in the Netherlands: a cross-sectional population-based serological survey. BMC Infect. Dis..

[40] Barnaud E., Rogee S., Garry P., Rose N., Pavio N. (2012). Thermal inactivation of infectious hepatitis E virus in experimentally contaminated food. Appl. Environ. Microbiol..

[41] Emerson S.U., Arankalle V.A., Purcell R.H. (2005). Thermal stability of hepatitis E virus. J. Infect. Dis..

[42] Yugo D.M., Meng X.J. (2013). Hepatitis E virus: foodborne, waterborne and zoonotic transmission. Int. J. Environ. Res. Public Health.

[43] Wichmann O., Schimanski S., Koch J., Kohler M., Rothe C., Plentz A., Jilg W., Stark K. (2008). Phylogenetic and case-control study on hepatitis E virus infection in Germany. J. Infect Dis..

[44] Bouwknegt M., Lodder-Verschoor F., van der Poel W.H., Rutjes S.A., de Roda Husman A.M. (2007). Hepatitis E virus RNA in commercial porcine livers in The Netherlands. J. Food Prot..

[45] Szabo K., Trojnar E., Anheyer-Behmenburg H., Binder A., Schotte U., Ellerbroek L., Klein G., Johne R. (2015). Detection of hepatitis E virus RNA in raw sausages and liver sausages from retail in Germany using an optimized method. Int. J. Food Microbiol..

[46] Giannini P., Jermini M., Leggeri L., Nuesch-Inderbinen M., Stephan R. (2018). Detection of hepatitis E virus RNA in raw cured sausages and raw cured sausages containing pig liver at retail stores in Switzerland. J. Food Prot..

[47] Cossaboom C.M., Heffron C.L., Cao D., Yugo D.M., Houk-Miles A.E., Lindsay D.S., Zajac A.M., Bertke A.S., Elvinger F., Meng X.J. (2016). Risk factors and sources of foodborne hepatitis E virus infection in the United States. J. Med. Virol..

[48] Alfonsi V., Romano L., Ciccaglione A.R., La Rosa G., Bruni R., Zanetti A., Della Libera S., Iaconelli M., Bagnarelli P., Capobianchi M.R. (2018). Hepatitis E in Italy: 5 years of national epidemiological, virological and environmental surveillance, 2012 to 2016. Euro. Surveill..

[49] Agence de l’eau Rhône Méditerranée Corse RAPPORT d’activité 2017. https://www.eaurmc.fr/jcms/pro_73733/fr/rapport-d-activite-2017.

[50] Iaconelli M., Purpari G., Della Libera S., Petricca S., Guercio A., Ciccaglione A.R., Bruni R., Taffon S., Equestre M., Fratini M. (2015). Hepatitis A and E viruses in wastewaters, in river waters, and in bivalve molluscs in Italy. Food Environ. Virol..

[51] Kokkinos P., Kozyra I., Lazic S., Soderberg K., Vasickova P., Bouwknegt M., Rutjes S., Willems K., Moloney R., de Roda Husman A.M. (2017). Virological quality of irrigation water in leafy green vegetables and berry fruits production chains. Food Environ. Virol..

[52] Maunula L., Kaupke A., Vasickova P., Soderberg K., Kozyra I., Lazic S., van der Poel W.H., Bouwknegt M., Rutjes S., Willems K.A. (2013). Tracing enteric viruses in the European berry fruit supply chain. Int. J. Food Microbiol..

[53] Brassard J., Gagne M.J., Genereux M., Cote C. (2012). Detection of human food-borne and zoonotic viruses on irrigated, field-grown strawberries. Appl. Environ. Microbiol..

[54] Kokkinos P., Kozyra I., Lazic S., Bouwknegt M., Rutjes S., Willems K., Moloney R., de Roda Husman A.M., Kaupke A., Legaki E. (2012). Harmonised investigation of the occurrence of human enteric viruses in the leafy green vegetable supply chain in three European countries. Food Environ. Virol..

[55] Ceylan A., Ertem M., Ilcin E., Ozekinci T. (2003). A special risk group for hepatitis E infection: Turkish agricultural workers who use untreated waste water for irrigation. Epidemiol. Infect..

[56] O’Hara Z., Crossan C., Craft J., Scobie L. (2018). First report of the presence of hepatitis E virus in scottish-harvested shellfish purchased at retail level. Food Environ. Virol..

[57] Crossan C., Baker P.J., Craft J., Takeuchi Y., Dalton H.R., Scobie L. (2012). Hepatitis E virus genotype 3 in shellfish, United Kingdom. Emerging Infect. Dis..

[58] Li T.C., Miyamura T., Takeda N. (2007). Detection of hepatitis E virus RNA from the bivalve Yamato-Shijimi (Corbicula japonica) in Japan. Am. J. Trop. Med. Hyg..

[59] Rivadulla E., Varela M.F., Mesquita J.R., Nascimento M.S.J., Romalde J.L. (2019). Detection of hepatitis E virus in shellfish harvesting areas from Galicia (Northwestern Spain). Viruses.

[60] Oliveira R., Mesquita J.R., Pereira S., Abreu-Silva J., Teixeira J., Nascimento M.S.J. (2017). Seroprevalence of hepatitis E virus antibodies in Portuguese children. Pediatr. Infect. Dis. J..

